# Altered immune signatures in breast cancer lymph nodes with metastases revealed by spatial proteome analyses

**DOI:** 10.1186/s12967-025-06415-4

**Published:** 2025-04-10

**Authors:** Oscar Briem, Balázs Tahin, Asger Meldgaard Frank, Lina Olsson, Anna Sandström Gerdtsson, Eva Källberg, Karin Leandersson

**Affiliations:** 1 Cancer Immunology, Department for Translational Medicine, Clinical Research Center, Lund University, Jan Waldenströms gata 35, Malmö, SE-214 28 Sweden; 2 Division of Clinical Pathology, Department of Clinical Sciences, Lund University, Malmö, 214 28 Sweden; 3 Division of Immunotechnology, Faculty of Engineering, Lund University, Malmö, 211 00 Sweden

**Keywords:** Breast cancer, Lymph node metastasis, CD169^+^ macrophages, B-cell follicles, Germinal centers, Interfollicular T-cells

## Abstract

**Background:**

Metastasis to lymph nodes is strongly associated with reduced survival in breast cancer patients. To increase the understanding on how lymph node metastasis impairs the local immune response in affected lymph nodes, we here studied spatial proteomic changes of critical lymph node immune populations in uninvolved lymph nodes (UnLN) and paired lymph nodes with metastases (LNM) from five breast cancer patients.

**Methods:**

The proteome was analyzed for cortical lymphocyte compartments, subcapsular sinus (SCS) and medullary sinus (MS) CD169^+^ macrophages, using the Digital Spatial Profiling (DSP) platform from NanoString.

**Results:**

Our results identified a stable proteome of SCS CD169^+^ macrophages in LNM, with the exception for downregulation of the anti-apoptotic protein Bcl-xL and FAPα, but a clear reduction in numbers of SCS CD169^+^ macrophages in LNM. In contrast, the proteome of MS CD169^+^ macrophages, B-cell compartments and interfollicular T-cells showed altered immune signatures in LNM, indicating that the decline in SCS CD169^+^ macrophages coincide with a malfunction in the local, anti-tumor immune responses.

**Conclusions:**

The findings from our study support the notion that metastasis to lymph nodes in breast cancer patients modifies local immune responses. These changes may contribute to explain unsuccessful therapeutic responses, and thereby worsened prognosis, for breast cancer patients with LNM.

**Supplementary Information:**

The online version contains supplementary material available at 10.1186/s12967-025-06415-4.

## Background

A major prognostic variable in breast cancer is whether the tumor has metastasized to sentinel lymph nodes (SLNs) or not [1]. SLN are typically the first site of metastasis, and patients with lymph node metastases (LNM), have a decreased overall and disease-free survival, irrespective of breast cancer subtype [1–3]. The spread of tumor cells to the draining lymph node leads to lymphangiogenesis, cytokine and chemokine trafficking, immune evasion, alterations in the tumor microenvironment and cancer cell expansion [4]. Which of these factors that play a pivotal role in tumor progression remains to be revealed. It has been debated whether this is mainly the result or measurement of a disseminated disease [5–7], or whether the lymph node metastasis actually modulates the local lymph node immune response, hence contributing to a worsened prognosis [5, 8, 9]. To be able to discuss clinical routines, therapeutical responses and future treatment options linked to lymph nodes of breast cancer patients, further research is needed to fully understand the details and impact of LNM on local lymph node immune responses. During disease progression, when compared to an uninvolved lymph node (UnLN i.e. non-metastatic), the anatomical structure of the LNM becomes disrupted [10]. Recent studies show that several features of the LNM microenvironment, including fibroblasts, amount of B-cell germinal center (GC) reactions and presence of certain immune cells, is linked to prognosis [11–14]. One such immune cell is the resident lymph node subcapsular sinus (SCS) CD169^+^ macrophage, which has been linked to a better patient prognosis when present in regional lymph nodes and LNM of various solid tumors [15–20], including tumor draining lymph nodes and LNM from breast cancer patients [21–24].

In lymph nodes there are various macrophages, but only two types are associated with residency and CD169 expression; the SCS CD169^+^ macrophages, located at the interface between the subcapsular sinus and B-cell follicles, and the medullary sinus (MS) CD169^+^ macrophages, located around the medullary cords [25]. Both SCS and MS macrophages reside at sites where they interact with the lymph, indicating their role in antigen processing and presentation. The location of SCS macrophages suggests a role as initiators of adaptive immunity. From studies in the mouse, it has been proposed that SCS macrophages may be critical for tumor antigen presentation in the draining lymph nodes, since they capture antigens from lymph fluid and present these to follicular dendritic cells or naïve B-cells, cross-present and finally activate CD8 T-cells [25, 26]. CD169^+^ SCS macrophages are also important for activation of T-cells in the cortical interfollicular regions (IFR) of lymph nodes [27, 28]. Although less is known about MS CD169^+^ macrophages, they exhibit higher lysosomal activity compared to SCS macrophages [29] and may help in clearance, or survival, of short-lived plasma cells [30, 31].

As mentioned above, SCS CD169^+^ macrophages, B-cells and interfollicular T-cells are connected both by spatial localization in lymph nodes and by initiation of an adaptive anti-tumor immune response [23, 26–28]. Development and maintenance of SCS macrophages is highly regulated by lymphotoxin-α1β2 which is expressed by B-cells [32] and B-cells are dependent on presence of SCS CD169^+^ macrophages for their expansion [23], partly explaining the importance of their co-localization. In the cortex of lymph nodes, in structures called B-cell follicles, naïve B-cells can be activated in either germinal center (GC)-dependent or -independent pathways [33]. Recently, a study showed that anti-tumor reactive B-cells can be derived from GC-independent B-cell follicles [34]. Interestingly, SLNs from breast cancer patients have distorted GC morphologies, with reduced number of GC in LNM compared to UnLN [13], indicating an effect on B-cell activation yet to be elucidated.

Since presence of SCS CD169^+^ macrophages in lymph nodes of cancer patients are associated with a better prognosis, it is important to understand their precise involvement in local anti-tumor immune reactions, to understand why they disappear from LNM and what impact this has on local lymph node immune reactivity. We previously studied CD169^+^ macrophages present in both primary breast tumors (PT) and LNM and found that they co-localize with B-cell containing tertiary lymphoid like structures in both locations [21, 24]. We further found that human monocyte derived CD169^+^ macrophages can enhance antibody production by activated B-cells, indicating an immune stimulating function with regards to B-cells also in humans [21, 24]. With an aim to progress the understanding of the role of these cells in the ongoing anti-tumor immune reactions occurring in lymph nodes of breast cancer patients, we here investigated the CD169^+^ macrophage- and B-cell compartments using spatial proteomics analysis in paired UnLN and LNM lymph nodes from five breast cancer patients.

## Methods

### Clinical samples and tissue processing

The study was conducted in accordance with the Declaration of Helsinki. Ethical approval was obtained by the Swedish Ethical Review Authority (Dnr 2021–04869). This study included whole sections of paired paraffinized uninvolved lymph nodes (UnLN) and lymph nodes with metastases (LNM) from five patients with invasive breast cancer. Only LNM with metastatic masses, rather than just diffuse cancer cells, were included. LNM in which metastatic masses comprised the majority of LNM were excluded to allow for proper visualization of lymph node structures. A clinical pathologist (B.T.) was responsible for evaluation and determination of inclusion/exclusion criteria. All primary tumors were estrogen (ER) and progesterone (PR) positive, with one patient also positive for human epidermal receptor-2 (HER2). NHG ranged from 1 (one case), 2 (three cases) to 3 (one case). Ki67 expression was high in 4 out of 5 primary tumors. All patients were untreated at the time for surgery. Samples were preserved in formalin-fixed paraffin-embedded (FFPE) blocks, sectioned at 4 μm thickness, and mounted on SuperFrost Plus IHC slides for subsequent analysis.

### Immunostaining

Presence of CD169^+^ macrophages and B-cells in all samples was first confirmed with immunohistochemistry (IHC) using anti-CD169 (clone SP216, Invitrogen, 1:100), Hematoxylin/Eosin (HE), or anti-CD20 (clone L-26, ThermoFisher Scientific, 1:200). Antigen retrieval and staining were conducted using a PT-link system (pH 9, K8010, DAKO/Agilent (Santa Clara, CA, US)) before staining with an Autostainer Link 48 system (DAKO/Agilent). Slides were scanned at 20X magnification using an Aperio Scanscope CS scanner (Leica Biosystems (Nussloch, Germany)) and further analyzed with QuPath 2.0 (see Data visualization and statistics). For fluorescent immunostaining of CD169, overnight staining was performed with rabbit monoclonal antibody (mAb) Anti-CD169 (clone SP216; Abcam 183356, 1:500) directly conjugated with AF647 (Invitrogen (Carlsbad, CA, US), A20186).

### GeoMx DSP technology

The NanoString Digital Spatial Profiler (DSP) GeoMx platform (Seattle, WA, US) was used to pre-process and collect data to investigate tissue heterogeneity and complexity. The GeoMx Protein Slide Preparation protocol was applied according to manufacturer’s description. GeoMx DSP uses morphological markers tagged with fluorophores to visualize the spatial localization of cells of interest. Primary antibodies against morphological and profiling markers (Supplementary Table 1) were applied overnight and nuclei was stained with SYTO13 prior to mounting slides in the NanoString DSP GeoMx instrument. Each slide was scanned, and regions of interest (ROIs) were selected. The scanned paired whole lymph node sections are shown in Supplementary Fig. 1 side by side. Using UV light, the UV-cleavable link attached to the profiling antibodies was disrupted, causing oligo barcodes from the ROIs to be collected in a 96-well plate, hybridized, and counted using an nCounter (NanoString). Our study included 96 ROIs representing three different tissue region types: abundance of CD45-positive cells; CD169-positive cells; and Pan-CK-positive cells, respectively. Subsequent data processing followed GeoMx standard workflow, including quality control based on field of view registration, binding intensity, positive control probe normalization, minimum nuclei count, and minimum surface area for a ROI.

### ROI strategy and optimal normalization of data

After quality control, normalization of the data was carefully evaluated to find the most suitable linear scaling normalization based on the geometric mean with either housekeeping proteins, negative isotype control antibodies, ROI area or nuclei count. All ROIs passed quality control and were included in the downstream analysis (Supplementary Fig. 2). The normalization method best suited for our data was determined to be scaling by Housekeeping proteins Histone 3 (H3) and ribosomal protein S6 which showed high correlation (Supplementary Fig. 2A-C). The geometric mean of these two markers was consequently used to scale the data. Signal-to-background ratios of each probe were consistently < 1 for three antibodies: Lag-3, CD80 and GITR. These markers were excluded from all further analysis (Supplementary Fig. 2D).

### Data visualization and statistics

QuPath was used for image analysis of IHC, where the output was the number of positive CD169 cell segments or CD20 cell segments in UnLN and LNM and image type was set to brightfield (H-DAB). IHC of paired whole lymph node sections are shown in Supplementary Fig. 3 (CD169) and Supplementary Fig. 4 (CD20 and CD169) side by side. Classification for CD169 positive cell segments was set with intensity threshold 0.8 (cell: DAB OD max), causing segmentation into cells either positive or negative for CD169. The percentage of positive CD169 cells were calculated from all cells present in UnLN and LNM, thereafter all CD169^+^ cells were divided into SCS or MS macrophages. Differences between UnLN and LNM were calculated with a paired student´s t-test using Graph Pad Prism 10. All spatial proteomics analysis were performed within the GeoMx DSP data analysis suite (V.3.1.0.194) from NanoString, including quality control assessments, data normalization and statistical analysis. Plots for data visualization were made with NanoString-validated R-scripts (GeoScript Hub, NanoString, available at https://nanostring.com/products/geomx-digital-spatial-profiler/geoscript-hub// (accessed 10 June 2023)), used to generate volcano plots, Principal Component Analysis (PCA) and hierarchical clustering analysis. For statistical analyses, Linear mixed model (LMM) adjusted for matched donors was used to compare protein expression between ROIs from UnLN to LNM ROIs, Paired t-test with Benjamini & Hochberg correction was used for SCS CD169 macrophages (where a ROI from each tissue was available) and Pearson correlation for supervised and unsupervised clustering of all ROIs in this study. Protein expression comparison was illustrated through Volcano plots, showing significantly (*P* < 0.05) differently expressed proteins. Forest plots indicating the distribution in ratio values between SCS and MS macrophages for macrophage markers were calculated from the relative expression of target probes in all macrophage ROI.

## Results

### Fewer SCS CD169^+^ macrophages in LNM compared to UnLN

**Fig. 1:**
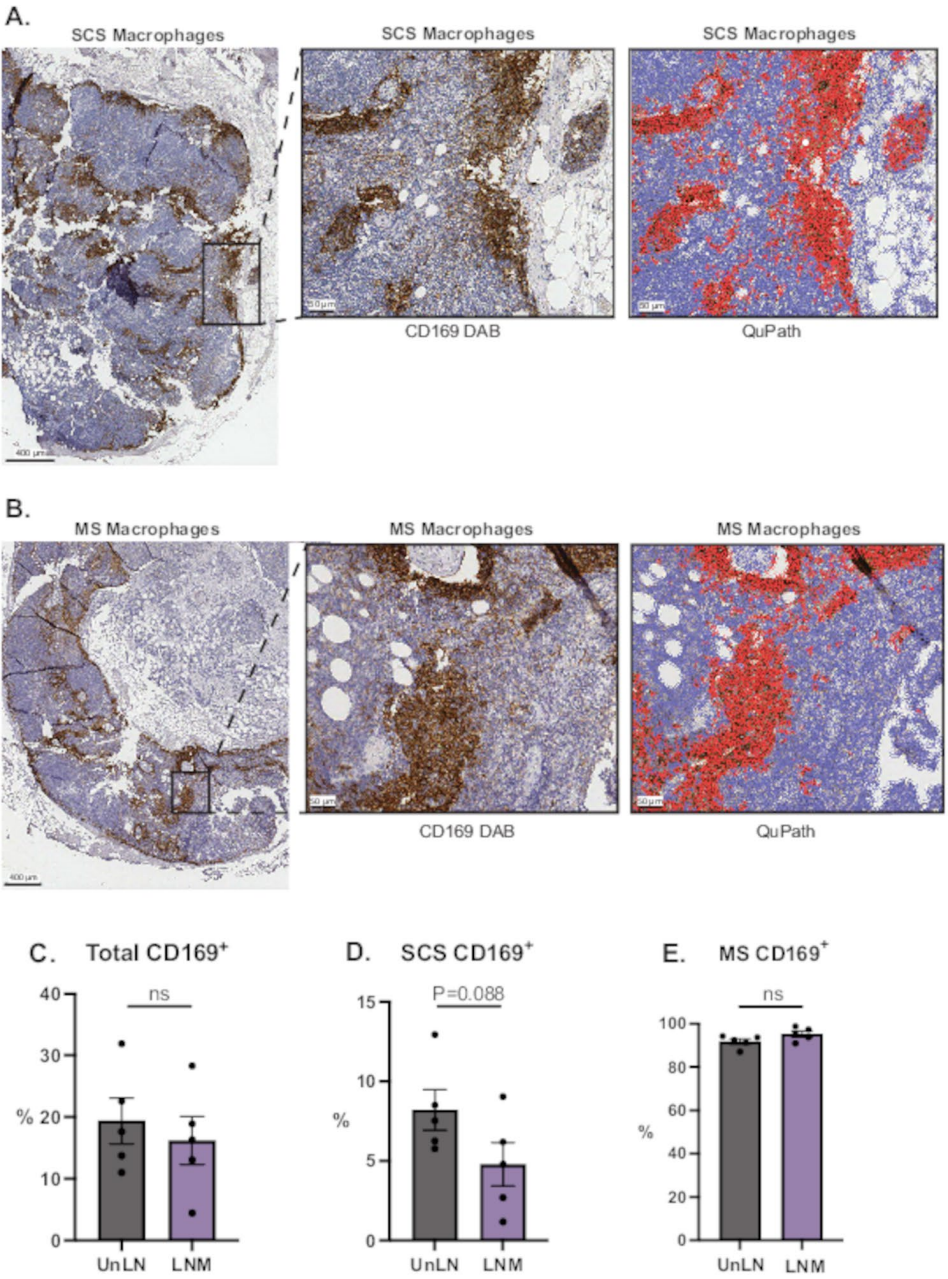
(**A-B**) Representative of breast cancer lymph node tissue stained with H&E and IHC (CD169) (**A**) SCS CD169^+^ macrophages and (**B**) MS CD169^+^ macrophages of whole lymph node sections from LNM together with zoomed visualizations of areas (DAB and segmentation in QuPath) (**C**) Quantification of bulk CD169^+^ cells (**D**) SCS CD169^+^ macrophages (**E**) MS CD169^+^ macrophages in UnLN compared to LNM using QuPath Software. Error bars indicate SEM, Statistics were performed with paired students T-test, * *P* < 0.01, ** *P* < 0.05, *** *P* < 0.001. *N* = 5

We included five paired lymph nodes from breast cancer patients and compared the LNM and UnLN. The criteria for LNM were that the metastasis should not cover a major part of the lymph node to be able to compare compartments and draw conclusions regarding immune cells. We initially investigated presence of CD169^+^ macrophages in the paired LNM/UnLN samples with the help of IHC and QuPath, to confirm previous knowledge regarding regression of CD169^+^ macrophages in involved lymph nodes from cancer patients [16, 21, 23, 29, 35, 36], but also to investigate their location as being SCS CD169^+^ macrophages or MS CD169^+^ macrophages. Representative IHC staining for SCS and MS macrophages are presented in Fig. 1A-B and whole sections of the paired lymph nodes are shown in Supplementary Fig. 3–4. Cells were visualized and classified using QuPath software, allowing positive cell detection for CD169^+^ macrophages (Fig. 1A-B). The effect of metastasis was investigated in the three subsets: All CD169^+^, SCS CD169^+^, and MS CD169^+^ macrophages. As shown in Fig. 1C, there was a trend towards fewer CD169^+^ macrophages in LNM in general. When dividing into SCS CD169^+^ and MS CD169^+^ macrophages based on spatial localization, a reduction specifically in SCS CD169^+^ macrophages was observed, whereas MS CD169^+^ macrophages were unchanged, or possibly slightly increased, in numbers in LNM compared to UnLN (Fig. 1D-E).

Hence, in LNM, the landscape of lymph nodes is disrupted with a reduction of SCS CD169^+^ macrophages in the subcapsular cortex, which eventually could lead to reduced interactions with underlying B-cell follicles and a weakened anti-tumor immunity. MS CD169^+^ macrophages were not affected.

### Clear data separation of ROIs reflected in GeoMX biomarker profiles

**Fig. 2:**
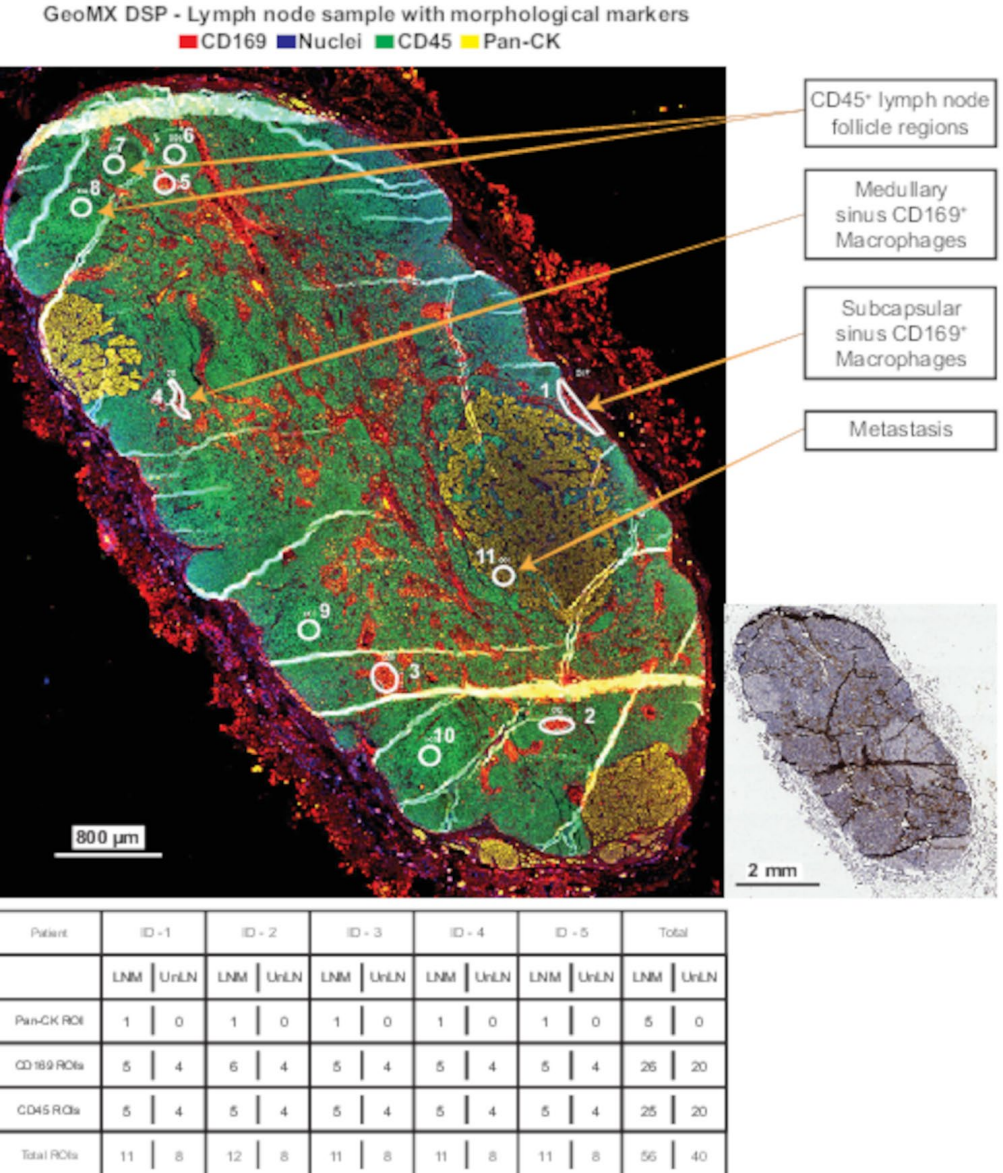
Visualization of ROI strategy applied to all samples. Four regions of cells were investigated with GeoMX DSP: Subcapsular CD169^+^ macrophages (ROI #1), Medullary sinus CD169^+^ macrophages (ROI # 2–5), CD45^+^ lymph node follicle regions (ROI #6–10), and the metastasis (ROI #11). ROIs from each lymph node are represented in the table below the figure

Using the GeoMx DSP platform, we next investigated proteome changes in CD169^+^ macrophage regions and cortical CD45^+^ lymph node follicle regions, in UnLN compared to paired LNM. This was done by staining for two specific biomarkers, CD169 for macrophages and CD45 for leukocytes in general, followed by spatial visualization, selecting CD45^+^ lymph node follicle regions localized in the cortex near the capsule of the lymph node and if possible, in close contact to SCS CD169^+^ macrophages. We also analyzed MS CD169^+^ macrophages as a separate population based on spatial localization in the MS. ROIs were selected to represent regions within the tissue containing the same type of immune cells (either CD45 alone, or CD45 and CD169), to enable comparison of the same types of cells in UnLN and LNM. The different types of ROIs represented SCS CD169^+^ macrophages, MS CD169^+^ macrophages, cortical CD45^+^ lymph node follicle regions and breast cancer metastatic cells stained with Pan-CK. Because SCS macrophages were reduced in LNM, ROIs representing MS macrophages were numerous in each sample. A schematic picture representing our ROI strategy is shown in Fig. 2. Whole sections of the stained paired lymph nodes are shown in Supplementary Fig. 1.

**Fig. 3:**
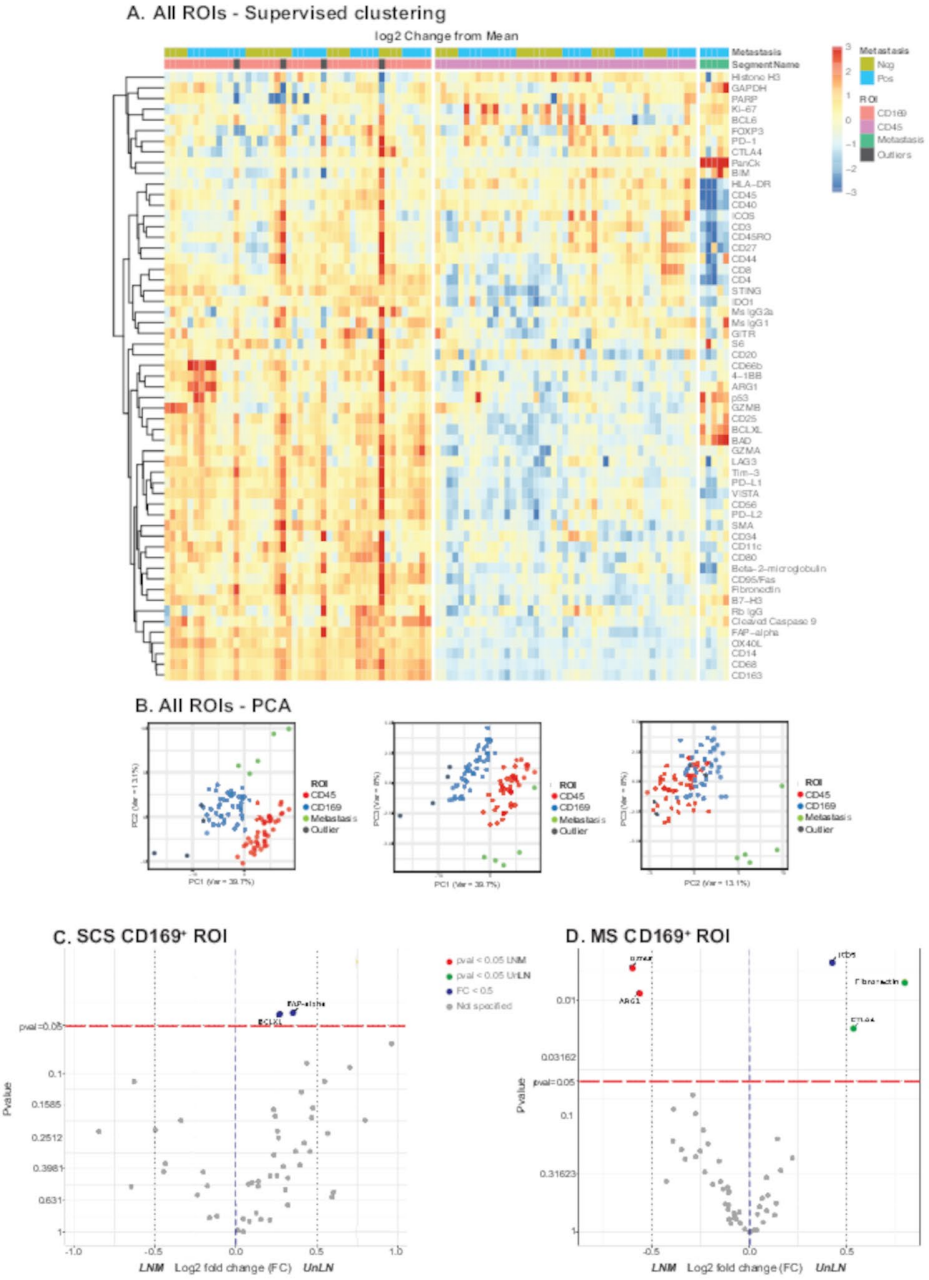
(**A**) General heat map with supervised clustering performed based on ROI. The color scale represents the log2 change from the geometric mean off all probes in the analysis, 4 outliers were excluded from CD169 ROI. Supervised clustering was performed based on ROI. (**B**) PCA plot representing all samples ROI illustrated with the first three principal components. Annotations: CD169 (macrophages), CD45 (lymphoid follicles), Metastasis (malignant cells metastasis), Outliers. (**C-D**) Volcano plots showing the statistical significance versus the magnitude of change in protein expression between five paired LNM (left) versus UnLN (right). Linear mixed model (LMM) adjusted for matched donors or paired T-test adjusted with Benjamini & Hochberg method was applied as statistical tests. Proteins with significant *P*-value < 0.05 are shown in blue, with proteins showing a log2 fold change above 0.5 plotted in red or green depending on the direction. (**C**) SCS CD169^+^ macrophage associated proteins with significant difference in expression between paired LNM and UnLN. (**D**) MS CD169^+^ macrophage associated proteins with significant difference in expression between paired LNM and UnLN

A heatmap of all ROIs illustrated the differential protein expression of CD169, CD45, tumor regions (Pan-CK) (Fig. 3A) and showed that ROIs representing CD45^+^ lymph node follicle regions, CD169^+^ macrophages and tumor cells, respectively, were accurately separated in the data. Concordantly, the CD169 ROIs had a high expression of typical macrophage markers such as CD14, CD68 and CD163, while CD45^+^ lymph node follicle ROIs had high expression of lymphoid markers. The tumor regions expressed Pan-CK, representing our control of metastatic breast cancer cells. The differences between each ROI type were also visualized with PCA, showing clear cluster separation (Fig. 3B). 4 ROIs from CD169 regions were considered as outliers based on the combined assessment from heatmap and PCA interpretation (indicated in grey in Fig. 3A-B) and excluded from the study.

### SCS and MS CD169^+^ macrophages show opposite trends in LNM

As presence of SCS CD169^+^ macrophages in LNM are associated with a beneficial prognosis, our aim was next to investigate their proteome in paired lymph nodes, but also to compare with the relatively undiscovered MS CD169^+^ macrophages. The cohort consisted of 46 CD169 ROIs, including four outliers which were excluded from the analysis. This resulted in 24 ROIs from LNM and 18 ROIs from UnLN. Our analysis was based on the localization of the CD169^+^ macrophage ROIs, i.e., SCS CD169^+^ macrophages versus MS CD169^+^ macrophages. Forest plots showing differential protein expression for SCS compared to MS macrophages in UnLN (Supplementary Fig. 5A) and LNM (Supplementary Fig. 5B) are shown to visualize potential macrophage polarization profiles. The proteome differences between SCS and MS CD169^+^macrophages, in LNM and UnLN respectively (Supplementary Figs. 5 and 6 A-B), showed that MS macrophages expressed more CD163, CD14, Cleaved Caspase 9 and CD68 in general. In LNM, MS macrophages showed an anti-inflammatory proteome polarization state or environment compared to SCS macrophages (CD163, OX40L, CTLA4, PD-L1/2, BAD, Bcl-xL, FoxP3, FAPα, SMA) (Supplementary Figs. 5 and 6 A-B). SCS macrophages expressed slightly more HLA-DR compared to MS macrophages in UnLN, although not significant, but not in LNM (Supplementary Fig. 5). SCS macrophages were in close contact with CD20^*+*^ cells representing B-cells, both in UnLN and LNM. This was in agreement with previous studies [37] and hence ROIs of SCS CD169^+^ macrophages versus MS CD169^+^ macrophages were confirmed.

Although SCS CD169^+^ macrophages decline in LNM, the remaining SCS CD169^+^ macrophages could obviously still have a functional role, or an altered function and hence impact on prognosis. Due to the fewer SCS CD169^+^ macrophages, our analysis consisted of 4 matched ROIs of SCS macrophages in each tissue type. Only two proteins showed a significant difference in expression comparing SCS CD169^+^ macrophages in UnLN and LNM: B-cell lymphoma-extra-large (Bcl-xL) and Fibroblast activation protein α (FAPα), with an overrepresentation in UnLN, or a downregulation in LNM of both proteins (Fig. 3C). The log2 fold change for both proteins of interest were below 0.5, indicating only minor changes in protein expression. Bcl-xL is an anti-apoptotic marker that regulates apoptosis. Its downregulation in LNM may therefore allude a potential mechanism explaining the disappearance of SCS macrophages in LNM. Importantly, apart from these two proteins, the rest of the proteome of the remaining SCS CD169^+^ macrophages remained unchanged, indicating similar functions for SCS CD169^+^ macrophages in UnLN as compared to LNM.

Next, we investigated the MS CD169^+^ macrophages in UnLN versus LNM. Due to their higher presence in all samples, a comprehensive analysis with 33 ROIs, 19 from LNM and 14 from UnLN was possible. For MS CD169^+^ macrophages ROIs, five proteins showed a significantly altered expression (Inducible costimulatory T-cell receptor (ICOS), Cytotoxic T-lymphocyte associated protein 4 (CTLA4), Fibronectin, Granzyme A (GZMA) and Arginase 1 (Arg1)) (Fig. 3D). While GZMA and Arg1 increased in expression, ICOS, CTLA4 and Fibronectin were downregulated in LNM as compared to UnLN.

In summary, our CD169 ROIs represent two populations of macrophages in lymph nodes; the declining SCS CD169^+^ macrophages with a stable proteome except for lower expression of the anti-apoptotic protein Bcl-xL and the enduring MS CD169^+^ macrophages with altered proteome in LNM.

### CD45 lymph node follicles separate into three distinct clusters

**Fig. 4:**
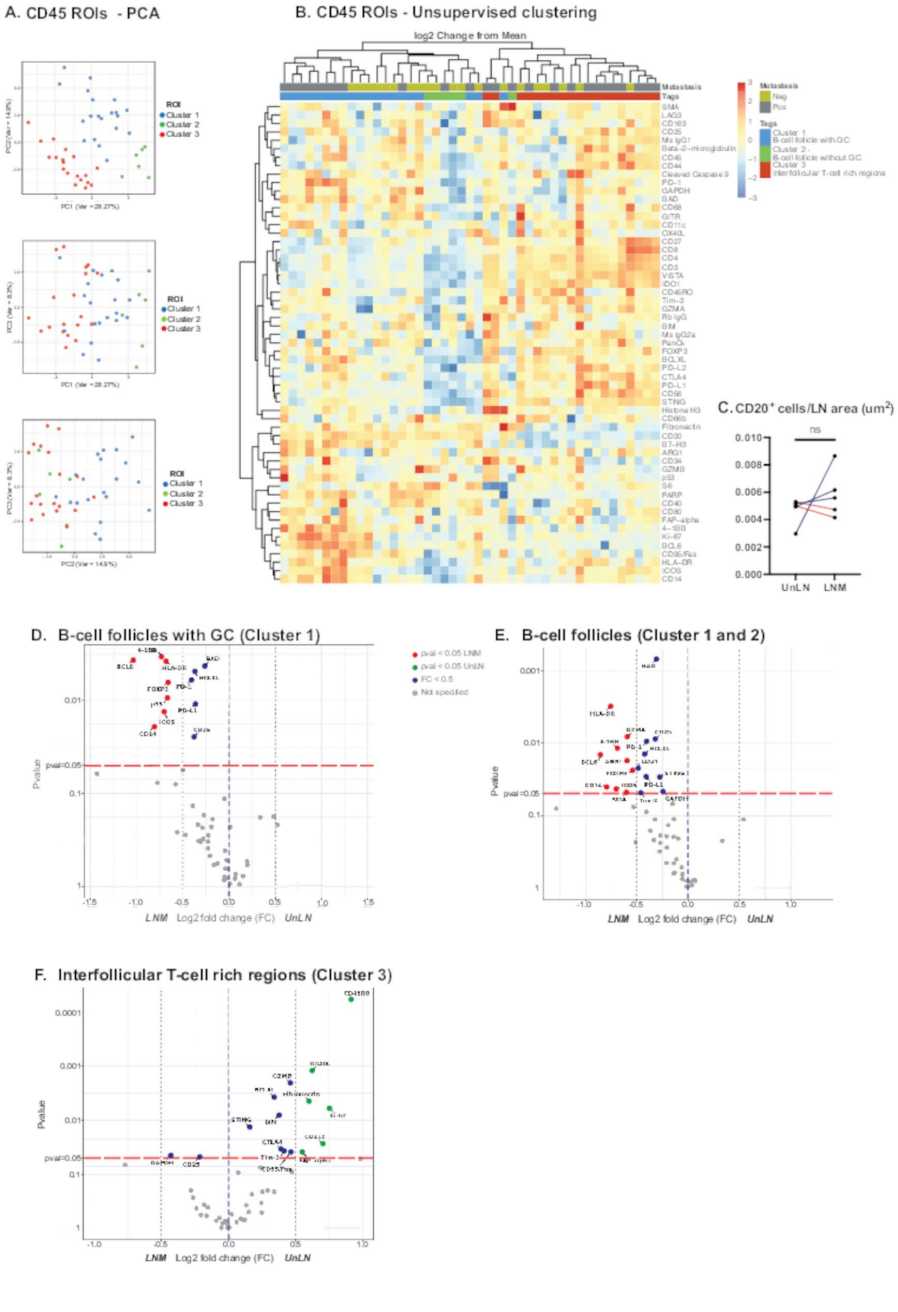
(**A**) PCA plot representing all CD45 ROIs illustrated with the first three principal components. Three clusters are shown. (**B**) General heat map with unsupervised clustering performed based on CD45 ROIs. The color scale represents the log2 change from the geometric mean off all probes in the analysis. Annotations: Neg (UnLN); Pos (LNM); B-cell follicles with GC (Cluster 1); B-cell follicles without GC (Cluster 2); Interfollicular T-cell rich region (Cluster 3). (**C**) Quantification of bulk CD20^+^ B-cells in UnLN compared to LNM using QuPath software. Statistics were performed with paired students T-test. (D-F) Volcano plots showing the statistical significance versus the magnitude of change in protein expression between five paired LNM (left) versus UnLN (right). Linear mixed model (LMM) adjusted for matched donors was applied as statistical tests. Proteins with significant *P*-value < 0.05 are shown in blue, with proteins showing a log2 fold change above 0.5 plotted in red or green depending on the direction (**D**) B-cell follicles with GC (Cluster 1) associated proteins with significant difference in expression between paired LNM and UnLN. (**E**) B-cell follicles associated proteins (Cluster 1 and 2) with significant difference in expression between paired LNM and UnLN. (**F**) Interfollicular T-cell rich region (Cluster 3) associated proteins with significant difference in expression between paired LNM and UnLN

As the SCS CD169^+^ macrophages are in spatial contact with B-cell follicles and MS CD169^+^ macrophages affect plasma cell survival and clearance, we next investigated how LNM impacted the cortical CD45^+^ lymph node follicle proteomes. ROIs with histology representing CD45 lymph node follicles were selected from the cortex near the SCS. Our cohort consisted of 45 ROIs with CD45 regions located in the cortex of lymph nodes, with typical B-cell follicle structures and morphology. 25 ROIs from LNM were compared to 20 ROIs from UnLN. With a PCA, data dimensionality revealed three distinct clusters (Fig. 4A), indicating that datapoints representing ROIs within one cluster are different from those in another cluster. To investigate the difference between these clusters, differential protein expression was visualized in an unsupervised cluster heat map (Fig. 4B). Cluster 1 represented B-cell follicles with an active GC as deducted by high expression of CD20, Ki-67 and B-cell lymphoma 6 (BCL6), Cluster 2 represented B-cell follicles rich in B-cells yet lacking a GC as deducted by expression of CD20, CD40 and lack of BCL6 and Ki-67; Cluster 3 represented a capsular proximal T-cell rich region, as deducted by higher expression of T-cells markers CD3, CD4 and CD8 and lower expression of CD40, CD20, BCL6 and Ki-67, possibly exemplifying interfollicular regions (IFR) of T-cells [27]. The location of ROIs representing Cluster 3 (ROI #8–10 in Fig. 2) were confirmed by consecutive sections and IHC, showing spatial localization of Cluster 3 in interfollicular areas (17 out of 19 ROIs representing Cluster 3; Supplementary Fig. 6C).

These clusters were respectively annotated as B-cell follicles with GCs (Cluster 1), B-cell follicles without GC (Cluster 2), and interfollicular T-cell rich regions (Cluster 3), all three which were present in both UnLN and LNM.

### Cortical lymphocyte regions have an altered immune signature in LNM

We next investigated how LNM impact the B-cell compartment. Hence, the B-cell follicle cluster proteomes were ultimately compared between LNM and UnLN. We initially analyzed the number of B-cells in LNM as compared to UnLN, by quantifying CD20^+^ cells by IHC and QuPath. As shown in Fig. 4C, no significant difference in B-cell numbers was found between LNM as compared to UnLN, indicating that the interdependence or synergistic decay in SCS CD169^+^ macrophages and B-cells seen in mice [23, 26], is not reflected in human breast cancer LNM.

For B-cells follicles with GCs (Cluster 1), 10 ROIs from LNM and 10 ROIs from paired UnLN were compared. Results showed a clear upregulation of several proteins involved in active GC reactions in LNM compared to UnLN (Tumor necrosis factor ligand superfamily member 9 (TNFSF9; 4-1BB), p53, BCL6, Bcl-xL, Bcl-2 associated agonist of cell death (BAD), Human Leukocyte Antigen DR isotype (HLA-DR), CD14, ICOS) but also proteins related to immune regulation or GC contraction (Forkhead box P3 (FoxP3), Programmed Cell Death Protein 1 (PD-1), Programmed Cell Death Ligand 1 (PD-L1) and CD25) (Fig. 4C).

ROIs for B-cell follicles without GC (Cluster 2) were scarce in our analyses (2 ROIs from LNM and 4 ROIs from UnLN) and therefore not possible to statistically analyze alone. Instead, B-cell follicles with and without GCs (Cluster 1 and 2) were combined, with 12 ROIs from LNM and 14 ROIs from UnLN. Cluster 1 and 2, showed a similar protein profile as B-cell follicles with GC, but with an additional upregulation of the proteins T-cell Ig- and mucin domain containing molecule-3 (TIM-3), Arg1, GZMA, Stimulator of interferon genes (STING), CD34, α-smooth muscle actin (SMA), GAPDH and loss of p53 in LNM (Cluster 1 & 2; Fig. 4E).

We finally analyzed the interfollicular T-cell rich regions (Cluster 3; Fig. 4F), comprising 13 ROI from LNM and 6 ROI from UnLN. Here, downregulation of several proteins in LNM were observed (CD45RO, Tumor necrosis factor receptor superfamily, member 4 (TNFRSF4; OX40L), Fibronectin, Ki-67, CD11c, FAPα, Bcl-2 Interacting Mediator of cell death (BIM), Bcl-xL, Granzyme B (GZMB), CTLA4, TIM-3, STING and CD95/Fas) and only two were upregulated in LNM (CD25 and GAPDH) (Fig. 4F). This indicates that critical immunoregulatory changes occur in interfollicular T-cell rich areas in human breast cancer LNM, indicating suppressed immune reactivity in interfollicular T-cells that previously have been shown to depend on CD169^+^ SCS macrophages [27].

In summary, in LNM of breast cancer patients, critical microenvironmental changes occur in B-cell follicles and interfollicular T-cell rich regions, a finding that possibly could be a consequence of the regression of SCS CD169^+^ macrophages and immunosuppressive MS CD169^+^ macrophages found in LNM.

## Discussion

Lymph node metastasis (LNM) is a critical prognostic indicator in various types of cancer [10]. However, the precise involvement of lymph nodes in tumor progression remains unclear. Specifically, it is uncertain whether lymph nodes facilitate metastatic invasion and spread of the tumor due to its structure and anatomical location, or if the immune response is suppressed, thereby permitting metastatic invasion [5–9]. To investigate the latter, we here performed spatial proteomics analyzing CD169^+^ macrophages and cortical lymphocytes in paired lymph nodes from breast cancer patients. Given the relatively limited knowledge regarding MS macrophages in LNM, we here focused on both SCS and MS CD169^+^ macrophages.

The lymph nodes included in this study were derived from patients with ER^+^ primary tumors. This is the tumor type that spreads most easily to SLN [38]. However, one of the tumors also had a HER2^+^ phenotype, representing a more aggressive breast cancer subtype also associated with SLN metastasis and worse prognosis [39]. We have previously shown that HER2-expression in primary breast tumors is significantly associated with CD169^+^PD-L1^+^ expression in the primary tumor, but not in LNM [15]. In this study we could also show that HER2 status was one of the variables affecting CD169^+^ LNM macrophages as prognostic marker in multivariable analyses [15], a finding that was not supported using a different breast cancer cohort [21]. Although the influence of the HER2 breast cancer subtype specifically on lymph node macrophages and immune responses remains unclear, different breast cancer subtypes indeed do affect macrophage polarization and function differently [40, 41]. However, evidence also suggests that macrophages may drive clinical subtype shifts [42, 43], also during LNM [44], a finding that warrants further investigation for understanding the relation between lymph node macrophages and breast cancer subtypes. In the study presented here, although we compare UnLN with LNM, a weakness is that both lymph nodes may still be affected to some extent by the primary tumor.

Consistent with previous research [16, 23, 29, 35, 36] our data show a decline in lymph node SCS CD169^+^ macrophages in LNM. We observed that this was linked to downregulation of the anti-apoptotic protein Bcl-xL and FAPα in LNM, while the rest of the proteome of SCS macrophage regions was unchanged. SCS CD169^+^ macrophages proposedly prevents metastatic niche development, as depletion of these macrophages in in vivo breast cancer models significantly increases metastatic burden [23]. The fact that the anti-apoptotic protein Bcl-xL was downregulated in the LNM SCS CD169^+^ macrophage regions, possibly reflects a cell death associated decline [45] induced by tumor cells in LNM. Alternatively, the decline may involve loss of interaction with fibroblast reticular cells expressing FAPα [46]. The specific loss of SCS CD169^+^ macrophages in LNM could nevertheless explain their prognostic impact. The reduced numbers of SCS CD169^+^ macrophages in LNM may also impair acquisition and presentation of tumor-antigens to follicular dendritic cells (FDCs), B-cells, or interfollicular T-cells, thus leading to a worse prognosis, as demonstrated in several studies [15, 18–21, 27, 47, 48]. However, as suggested by our results, apart from Bcl-xL and FAPα, the remaining SCS CD169^+^ macrophages in LNM do not have an altered phenotype, hence indicating an unaltered function per se compared to UnLN, a finding that needs further investigation.

In contrast, an altered immune signature including increased activity of immunosuppressive Arg1, was found in LNM areas with MS CD169^+^ macrophages. Also, the numbers of MS CD169^+^ macrophages in LNM were preserved or even slightly increased. It has previously been shown that MS CD169^+^ macrophages may help in clearance or survival of short-lived plasma cells [30, 31], but also to regulate tolerance by phagocytosis of antigen specific T-cells [49]. Our results point in the direction that MS CD169^+^ macrophages likely interact closely with T-cells, evidenced by detection of T-cell related proteins (ICOS, CTLA4 and GZMA). We show that in LNM MS CD169^+^ macrophage areas, CTLA4 and ICOS were downregulated, while GZMA and Arg1 were upregulated. Downregulation of CTLA4 and ICOS in the LNM MS CD169^+^ macrophage areas could indicate both immunogenic and immunosuppressive events, possibly affecting anti-tumor T-cell activity in LNM [50, 51]. The higher expression of Arg1, a potent inhibitor of T-cell responses [52], in LNM MS CD169^+^ macrophage areas would support the latter. Furthermore, GZMA expressed by T-cells [53, 54], can in its extracellular form be internalized by macrophages leading to the secretion of pro-inflammatory mediators, thereby amplifying the inflammatory milieu in the MS macrophage regions [55, 56]. This has been shown to contribute to extracellular matrix (ECM) remodeling by degrading fibronectin [57], consistent with the observed reduced expression of fibronectin in LNM in this study. In summary, for lymph node CD169^+^ macrophages in breast cancer patients, our data indicate that LNM enables a metastatic niche inducing loss of SCS CD169^+^ macrophages and change of MS CD169^+^ macrophage phenotype into more immunosuppressive cells, hence modulating the local lymph node anti-tumor immune milieu. Indeed, although not significant, immune activating HLA-DR was expressed at higher levels on SCS CD169^+^ macrophages compared to MS CD169^+^ macrophages only in UnLN, indicating severe changes in LNM. Comparing the proteome of SCS and MS CD169^+^ macrophages, also enable evaluation of our previous findings regarding tumor infiltrating CD169^+^ macrophages in primary breast tumors [24], indicating that these could be more similar to MS CD169^+^ macrophages than to SCS CD169^+^ macrophages.

Anti-tumor B-cell responses is an emerging research area and the impact of LNM on B-cell activation is essential for understanding adaptive immune responses against metastasis. Our cohort analysis identified two B-cell clusters: B-cell follicles with active GCs and B-cell follicles without active GCs, each cluster present in UnLN as well as LNM. Expression of the proteins ICOS, BCL6, BAD, p53, 4-1BB, CD14, HLA-DR, PD-1 and CD25 in B-cell follicles with active GCs, shows that GCs are clearly activated in LNM compared to UnLN [58–68]. However, the higher expression of FoxP3, CD25, PD-1, PD-L1 and BCL6, may also indicate functional changes, including premature GC shutdown with reduced generation of long-lived plasma B-cells in LNM [69], or possibly a microenvironment submitted to T_regs_ and high immunosuppressive signals [70–72]. Indeed, initiation of GC shutdown has previously been shown to involve both T_FH_ cells expressing FoxP3, PD-1 and BCL6 [70–72] and ICOS-expressing T_regs_ that inhibit anti-tumor reactions [73, 74]. When adding B-cell follicles without GCs to the analyses, STING was upregulated in LNM, congruent with recent findings that interferon response promotes metastasis [8], but also additional immunoregulatory proteins (e.g. Arg1 and Tim-3) had increased expression. Taken together, this indicates that LNM promotes an immunoregulatory microenvironment in active B-cell follicles.

Finally, we also identified a capsular proximal T-cell rich cluster in the follicle areas, possibly representing interfollicular T-cells. Our data implied that LNM affecting the decay of SCS CD169^+^ macrophages occurred in parallel with a reduced interfollicular T-cell-expansion, as evidenced by significantly lower Ki-67 expression and T-cell markers in Cluster 3 (e.g. CD45RO, OX40L, GZMB, CTLA4, Tim-3) in LNM compared to UnLN. These data support previous literature where interfollicular T-cells are dependent on presence of SCS CD169^+^ macrophages for their expansion [27]. In contrast to previous studies on an interdependence or synergistic decay in SCS CD169^+^ macrophages and B-cells seen in mice [23, 26, 32], we did not find a reduced B-cell compartment in human breast cancer LNM. These data is in line with a recent study [36]. Furthermore, the data point in the direction that T-cell activation is affected in local breast cancer lymph node immune responses, also supporting the recent finding in other cancer forms [75]. Lastly, our analysis showed proteome changes representing other cell populations like fibroblasts and fibroblast reticular cells (FRCs), which could contribute to the formation of the LNM niche. FRCs regression facilitates structural lymph nodes changes, promoting metastasis progression and warrants further investigation.

## Conclusions

In conclusion, our study shows that breast cancer patients with lymph node metastasis have a compromised local immune environment in LNM as compared to UnLN. These changes most likely cause a dysregulated local anti-tumor immune response in LNM. Our findings underscore the critical role for CD169^+^ macrophages in maintaining lymph node integrity and immune responses during metastasis, affecting both the T-cell and B-cell compartment. Our data emphasize the need for further research to understand and mitigate the immune suppression observed in LNM, to predict treatment responses to immunotherapy and clinical strategies regarding lymph node surgery.

## Electronic supplementary material

Below is the link to the electronic supplementary material.

Supplementary Figure 1–6 and Supplementary Table 1 (Suppl files Briem et al. JTM.pdf)

## Data Availability

Available from the authors upon request.

## Abbreviations

4-1BB: Tumor necrosis factor ligand superfamily member 9
APC: Antigen presenting cell
Arg1: Arginase 1
BCL6: B-cell lymphoma 6
Bcl-xL: B-cell lymphoma-extra-large
BIM: Bcl-2 Interacting Mediator of cell death
CTLA4: Cytotoxic T-lymphocyte associated protein 4
DC: Dendritic cell
ECM: Extracellular matrix
ER: Estrogen receptor
FAPα: Fibroblast activation protein α
FFPE: Formalin-fixed paraffin embedded
GC: Germinal Center
GZMA: Granzyme A
GZMB: Granzyme B
H3: Histone 3
HE: Hematoxylin-eosin
HER2: Human epidermal receptor-2
HK: Housekeeping proteins
HLA-DR: Human leukocyte Antigen DR isotype
ICOS: Inducible costimulatory T-cell receptor
IFR: Interfollicular region
IHC: Immunohistochemistry
LMM: Linear mixed model
LNM: Lymph node metastasis
MS: Medullary sinus
OX40L: Tumor necrosis factor receptor superfamily, member 4
Pan-ck: Pan-cytokeratin
PCA: Principal Component Analysis
PD-1: Programmed Cell Death Protein 1
PD-L1: Programmed Cell Death Ligand 1
PR: Progesterone receptor
ROI: Region of interest
SCS: Subcapsular sinus
SEM: Standard error of the mean
SLN: Sentinel lymph node
SMA: α-smooth muscle actin
STING: Stimulator of interferon genes
T_FH_: Follicular helper T-cell
TIM-3: T-cell Ig- and mucin domain containing molecule-3
Tpex: T-cell progenitor exhausted cell
T_reg_: Regulatory T-cell
UnLN: Uninvolved Lymph Nodes
