# Supplementary material for: Altered immune signatures in breast cancer lymph nodes with metastases revealed by spatial proteome analyses
Source: J Transl Med. 2025 Apr 10;23:422. doi: 10.1186/s12967-025-06415-4 (PMC11987258; doi:10.1186/s12967-025-06415-4)
Supplement: Supplementary file 1 — Supplementary Figure 1–6 and Supplementary Table 1 (Suppl files Briem et al. JTM.pdf) [file 12967_2025_6415_MOESM1_ESM.pdf]

Supplementary Figure 1

Whole lymph node sections DSP GeoMx analysis

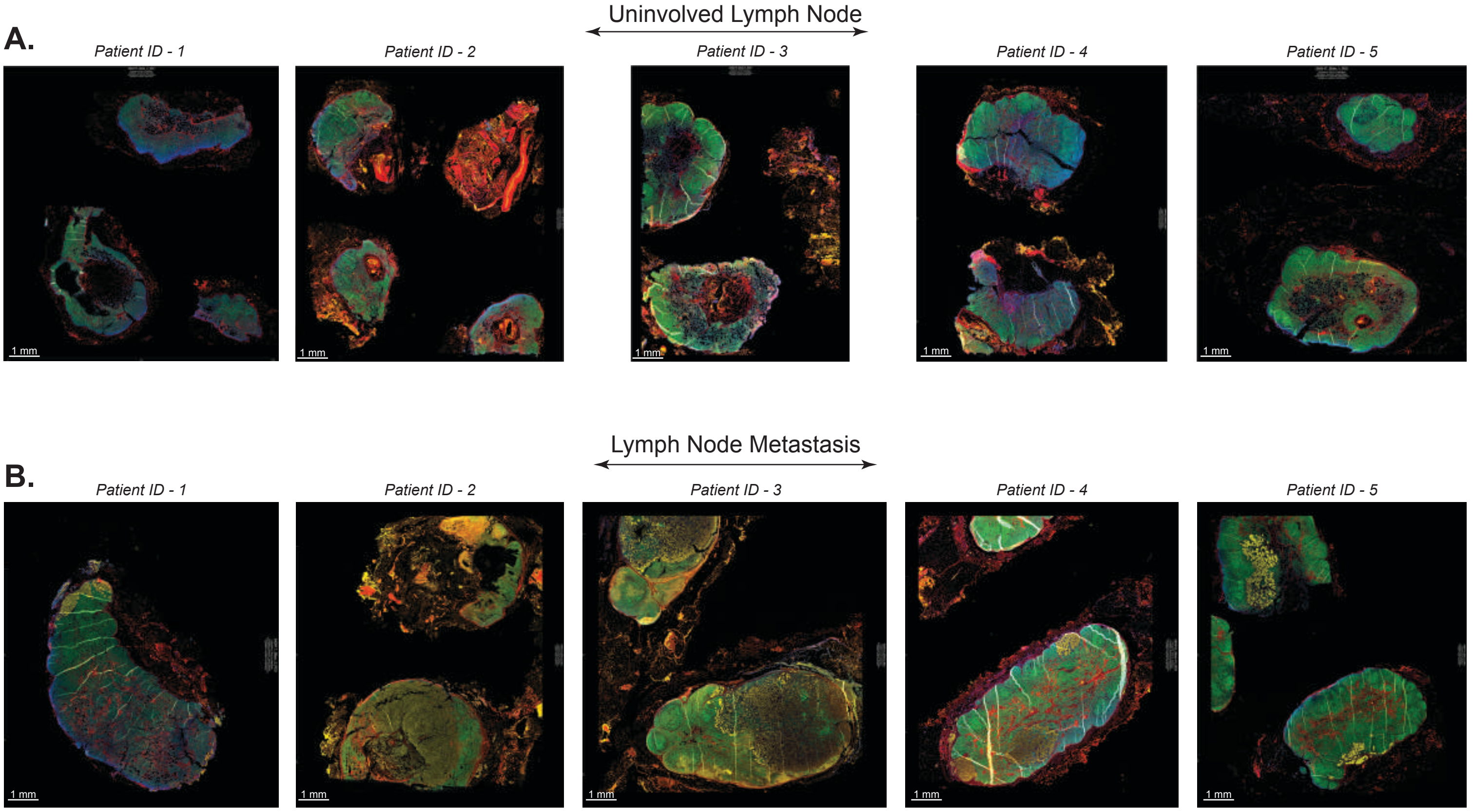

Supplementary Figure 1

Paired whole lymph node sections without (UnLN) and with (LNM) metastasis from five breast cancer patients stained with pan-leukocyte CD45 (green), Pan-CK for malignant cells (yellow) and CD169 (red) in the GeoMX DSP platform.

Supplementary Fig. 2

A. Normalization plots - Background

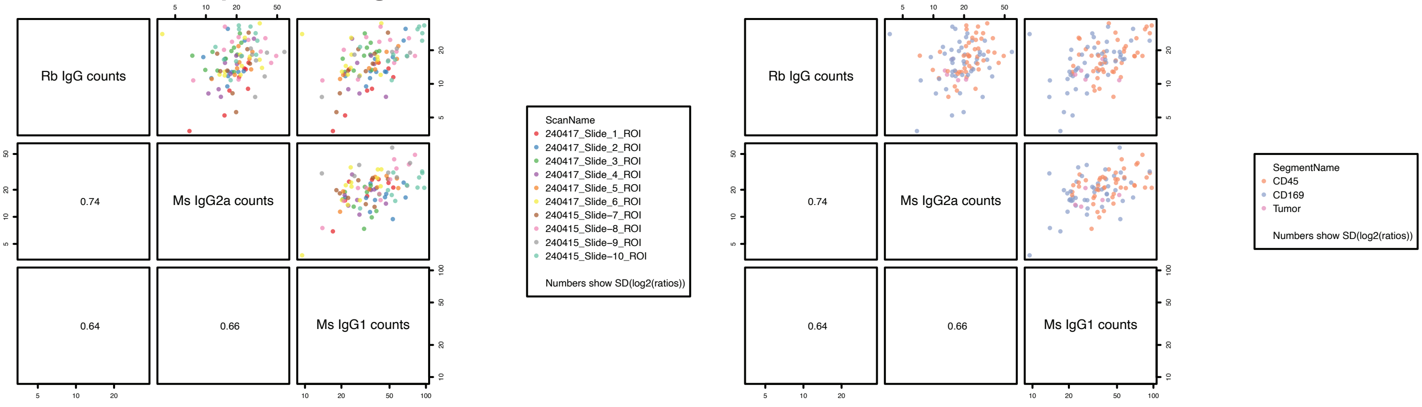

B. Normalization plots - Area and nuclei scaling

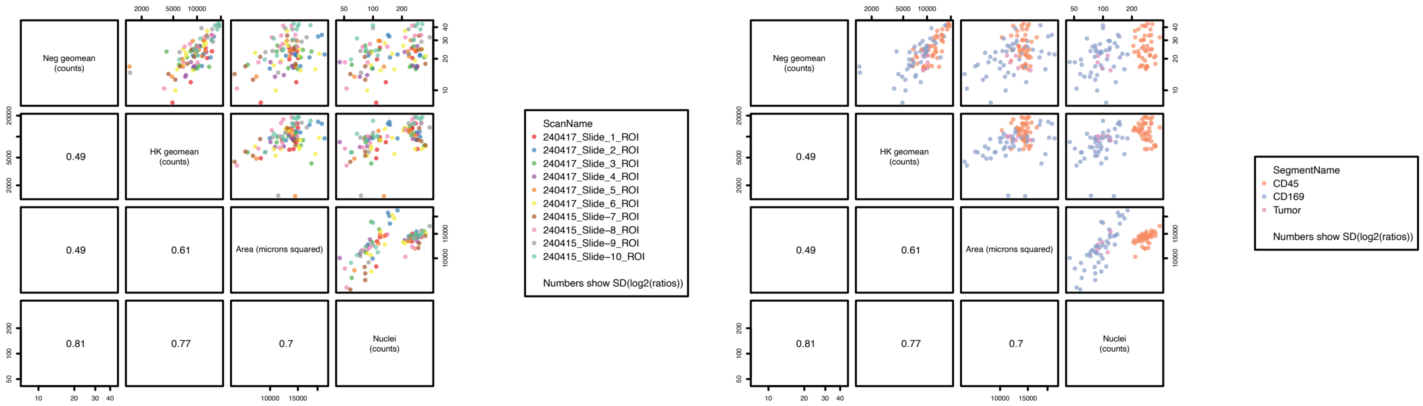

C. Normalization plots - House keepers

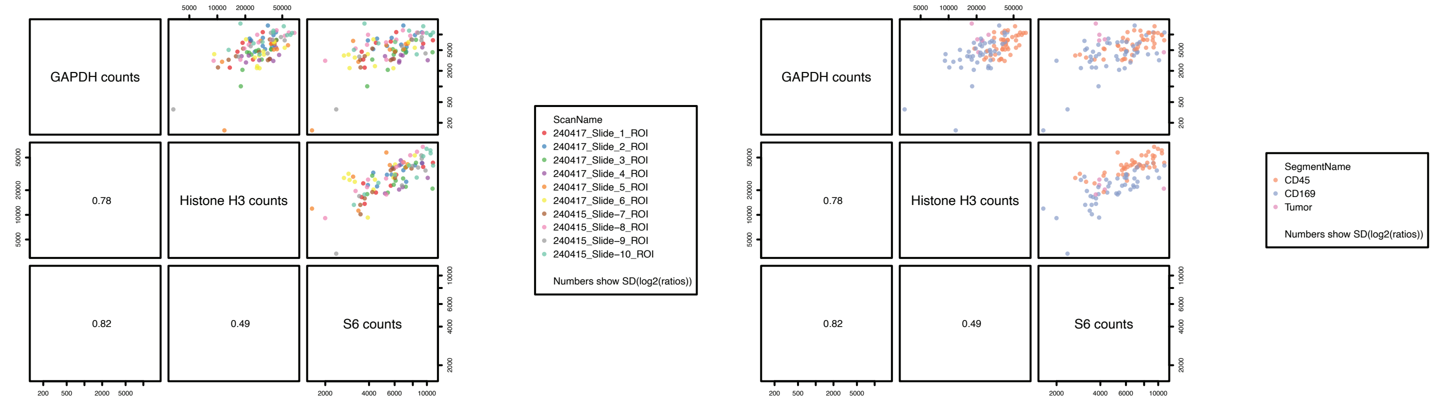

D. Signal background for each probe

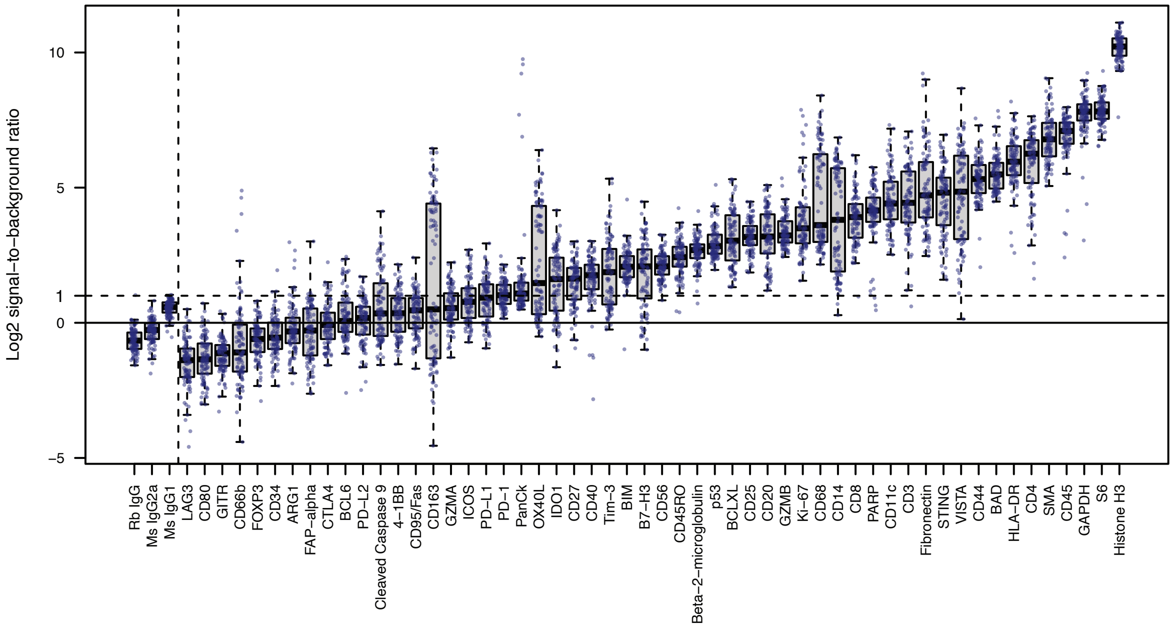

Supplementary Figure 2

Normalization strategy used after quality control (QC), concordance between each probe is shown by standard deviation of the log ratios between the geometric means. (A) Pairwise correlation plots of background probes from the dataset. Background isotype controls revealed weak correlations and low geometric mean counts, thus indicating statistical instability and not suitable as a normalization factor (B) Pairwise correlation plots of background, housekeeper, area scaling or nuclei scaling from the dataset. Due to variation in cell size and cell density between macrophages, lymphocytes and tumor cells, nuclei counts and ROI area displayed high variability between ROIs and low correlation with housekeeping probes and background signaling, thus not best suited for normalization (C) Pairwise correlation plots of housekeeper probes from the dataset. Housekeeping probes showed strong correlation and high statistical stability, with the best suited correlation observed between housekeeping proteins Histone 3 (H3) and ribosomal protein S6. (D) Signal background from each probe in the dataset, CD80, LAG-3 and GITR were excluded from the analysis due to lower signal as compared to background probes (left).

Supplementary Figure 3

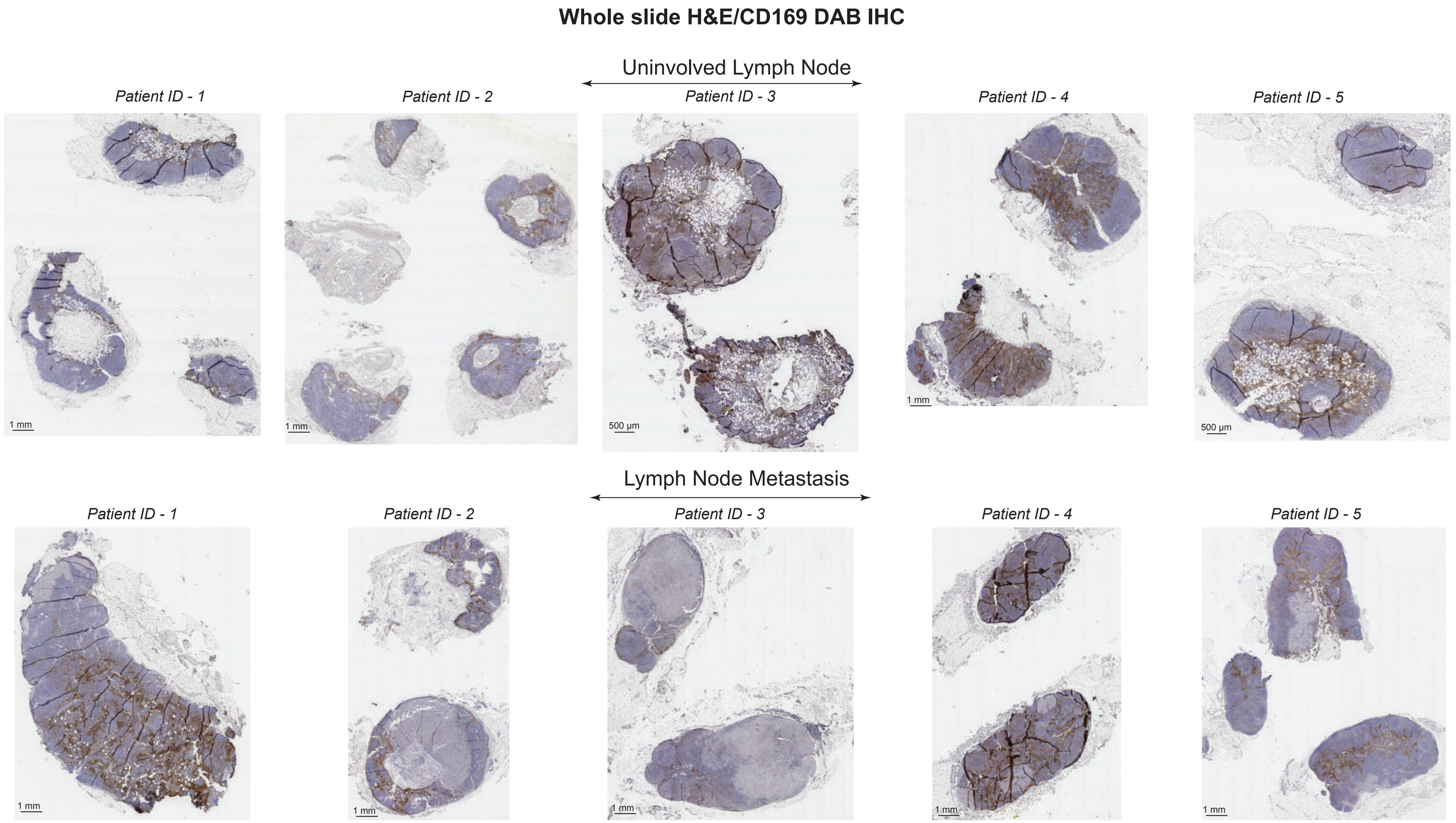

Supplementary Figure 3

Paired whole lymph node sections without (UnLN) and with (LNM) metastasis from five breast cancer patients stained with H&E and IHC (CD169 brown).

Supplementary Figure 4

Whole slide CD169/CD20 IHC

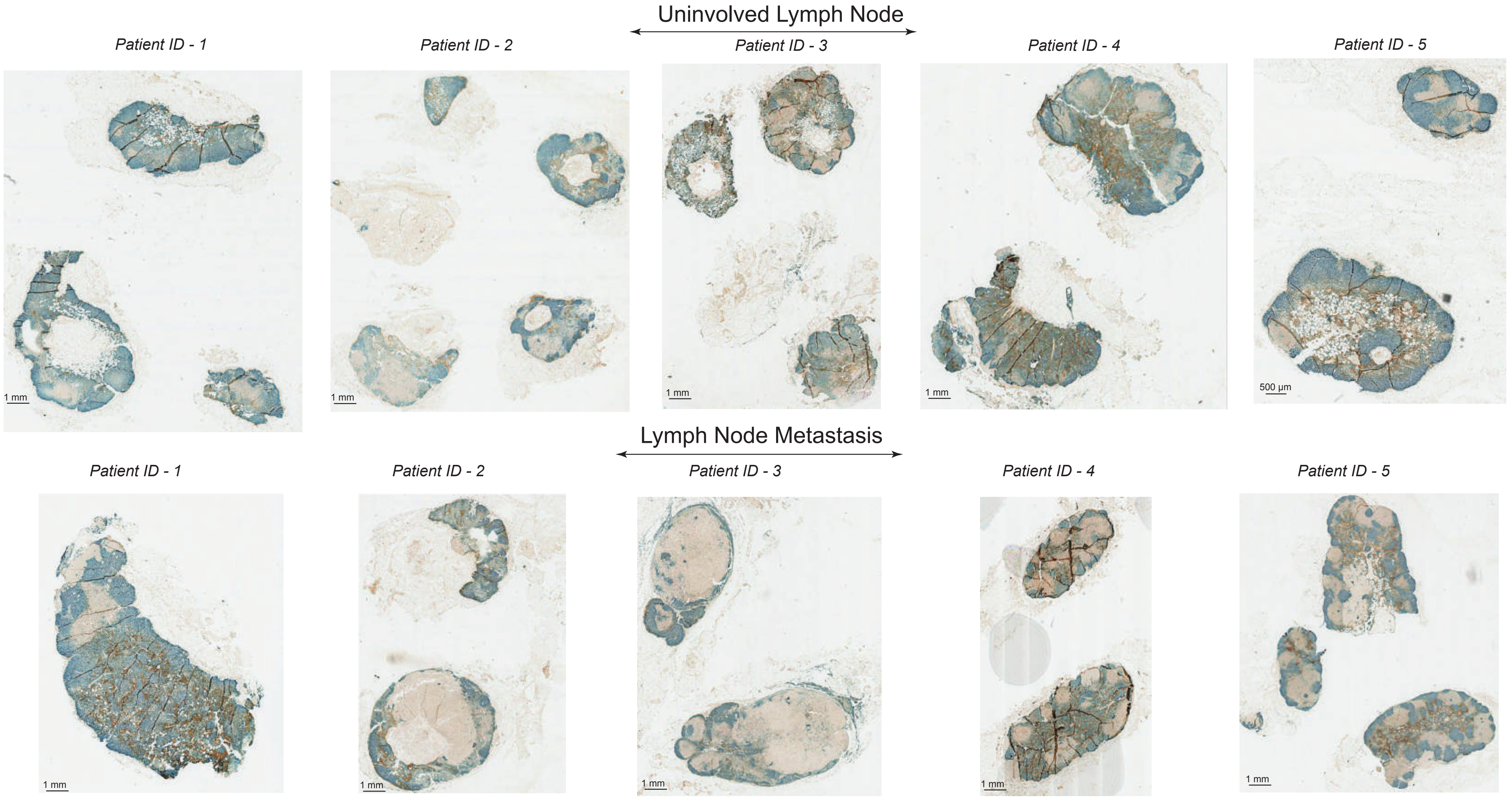

Supplementary Figure 4

Paired whole lymph node sections without (UnLN) and with (LNM) metastasis from five breast cancer patients stained with H&E and IHC (CD20 (blue) and CD169 (brown)).

Supplementary Figure 5

Differences in expression levels for selected proteins SCS CD169$^+$ vs MS CD169$^+$ macrophages in UnLN and LNM

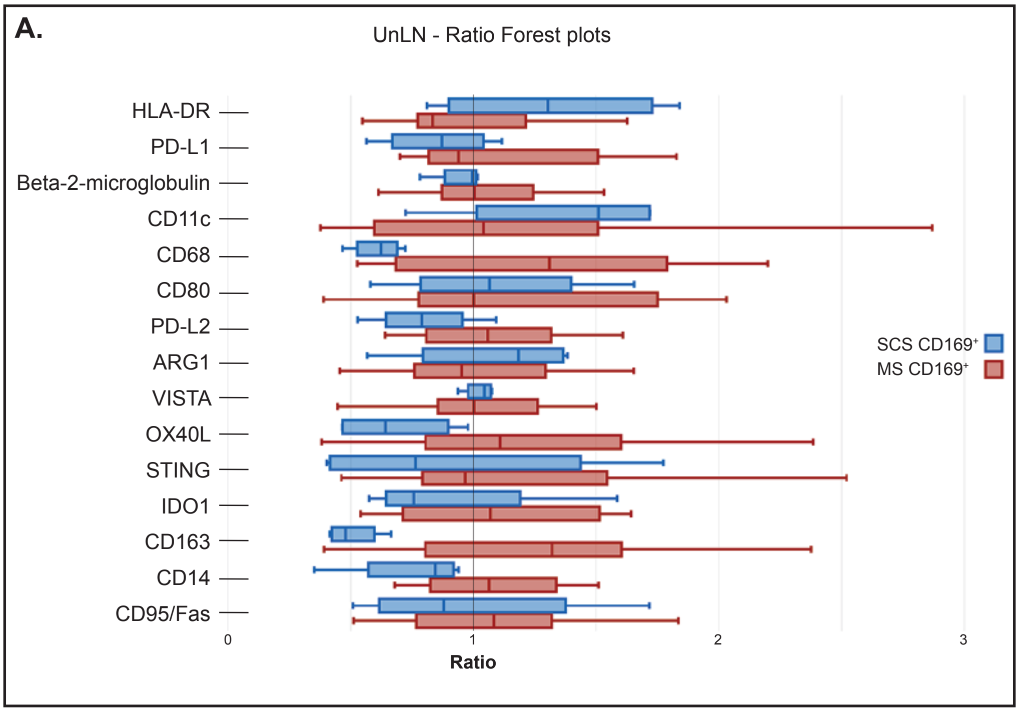

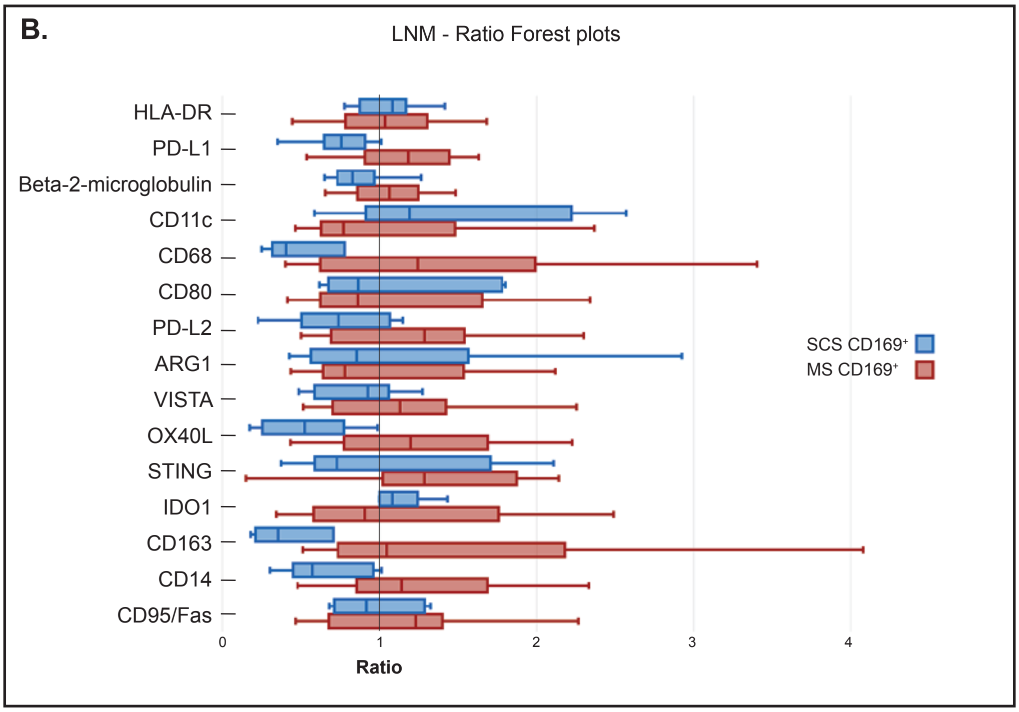

Supplementary Figure 5

Forest plots presenting selected proteins with differences in protein expression levels for SCS CD169$^+$ macrophages (blue) versus MS CD169$^+$ macrophage (red) in UnLN (A) and LNM (B).

Supplementary Figure 6

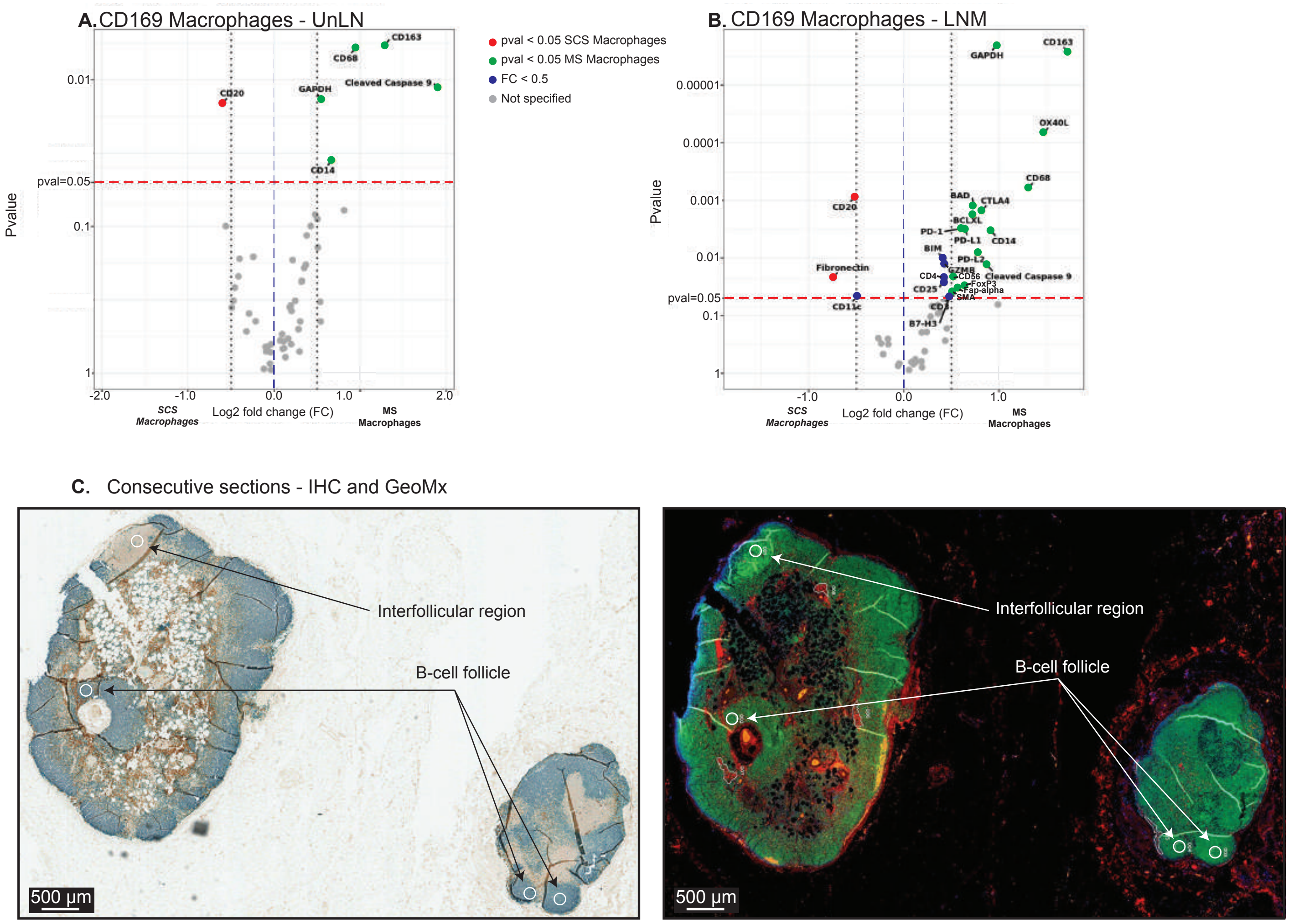

Supplementary Figure 6

(A-B) Volcano plots representing statistical significance versus the magnitude of change in protein expression for SCS CD169^{+} macrophages (left) versus MS CD169^{+} macrophage (right) in UnLN (A) and LNM (B). (C) anti-CD20 IHC of a consecutive section from one representative patient UnLN. IHC (left) shows that the location of cortical CD45^{+} lymph node follicle ROIs used in GeoMX (right) are present in both cortical CD20^{+} regions representing B-cells follicles and in CD20^{-} regions representing interfollicular region (IFR) T-cell areas.

Supplementary Table 1: Antibodies used for GeoMX DSP analysis

Abs/reagent	Type	Clone	Ab Dilution	DSP channel - EM max
Anti-CD169	Morphological marker	SP216	1:50	Cy5 – 666 nm
Anti-CD45	Morphological marker	NanoString	1:200	Cy3 – 568 nm
Anti-PanCK	Morphological marker	NanoString	1:1000	Texas Red – 615 nm
SYTO13	Morphological marker	NanoString	500 nM	FITC – 525 nm
Core Abs – Cell profiling	Profiling marker	NanoString	8 µl in 200 µl	None
Module Abs – Drug Target	Profiling marker	NanoString	8 µl in 200 µl	None
Module Abs - Immune activation	Profiling marker	NanoString	8 µl in 200 µl	None
Module Abs – Cell Death	Profiling marker	NanoString	8 µl in 200 µl	None
Module Abs - Cell typing	Profiling marker	NanoString	8 µl in 200 µl	None
